# Modification of Polylactide/Poly(Methyl Methacrylate) Blend Properties by Reduction in Macromolecular Entanglements

**DOI:** 10.3390/ma19163409

**Published:** 2026-08-11

**Authors:** Justyna Krajenta, Andrzej Pawlak

**Affiliations:** Centre of Molecular and Macromolecular Studies, Polish Academy of Sciences, Sienkiewicza 112, 90-363 Lodz, Poland; justyna.krajenta@cbmm.lodz.pl

**Keywords:** polylactide, poly(methyl methacrylate), miscible blend, macromolecular entanglements, crystallization, spherulites

## Abstract

**Highlights:**

- PLA/PMMA miscible blends having reduced density of entanglements were produced by extrusion.
- The limitation of macromolecular entanglements resulted in a reduction in the rheological moduli of the blend.
- Disentangling of macromolecules resulted in faster crystallization of PLA in the blend.

**Abstract:**

Polylactide (PLA) having partially disentangled macromolecules was blended by extrusion with an equilibrium-entangled poly(methyl methacrylate) (PMMA). Partial disentanglement of PLA macromolecules was obtained by dissolving the polymer in methylene chloride, freezing the diluted solution, and removing the solvent. Blends with PLA macromolecules with three levels of entanglements, containing 10, 20 or 80% of PMMA, were characterized using rheological studies and by conducting non-isothermal and isothermal crystallization. Scanning electron microscopy showed that only a small fraction of PMMA did not disperse at the molecular level. The blends prepared using less entangled PLA had lower values of rheological moduli and viscosity, which confirmed the limited density of macromolecular entanglements in them. It was found that the crystallization of PLA in blends was controlled by two main factors: the disentanglement of PLA macromolecules, which promoted faster crystal formation, and the presence of PMMA macromolecules, which hindered the movement of PLA macromolecules to crystal growth sites, making crystallization more difficult. Spherulites grew in blends containing 0–20% PMMA. PMMA macromolecules were incorporated into their structure.

## 1. Introduction

Macromolecules of most polymers, due to their length and flexibility, easily become entangled with other macromolecules [1]. The network of entangled macromolecules formed in this way—in an amorphous polymer or in the amorphous phase of a semicrystalline polymer—determines a number of the polymer’s properties in the solid and liquid state [2]. It is assumed that in polymers a certain equilibrium entanglement density is formed, characteristic of the type of polymer [3,4]. It can be determined from rheological studies, or based on mechanical properties. Macromolecules, depending on the temperature, perform more or less intense movements, which means that their entanglements are not constant; some disappear while others appear [5]. It is difficult to experimentally track the movement of a single macromolecule among others with which it is entangled, but models have been developed that allow linking the macroscopic properties of the polymer with the motion of the macromolecules. The most developed model is the tube model, in which the polymer chain moves inside a virtual tube formed by other molecules [6]. The movement of the polymer resembles a snake slithering and is called reptation, and the time it takes for the chain to move along the length of the tube is called the reptation time [7].

The basic relationships between the entanglements of macromolecules and rheological [8], mechanical properties [9], or the ability to crystallize have been determined based on studies of equilibrium-entangled homopolymers [10]. It is known, for example, that a network of entangled macromolecules affects the force that must be applied to deform the polymer during mechanical tests [11]. The values of rheological moduli should also depend on entanglements. Finally, in the crystallization process, it is more difficult for a macromolecule to reach the crystallization site if the polymer is more entangled [12].

To verify the correctness of the conclusions drawn from the above experiments, methods were developed to limit the density of entanglements of macromolecules, even several times less [5]. The most commonly used approach on a laboratory scale is based on observations that in very dilute polymer solutions, it is possible to achieve even the separation of individual macromolecules. If such a solution is frozen, and then the solvent is removed by sublimation, a polymer in a solid state is obtained, with disentangled macromolecules [10,12,13,14]. In practice, the density of entanglements is only partially limited in order to maintain the continuity of the polymer material, and therefore, for example, the possibility of studying mechanical properties. A number of partially disentangled polymers have already been studied, and the studies confirmed the importance of the entanglement network of macromolecules [14,15,16,17,18,19,20]. Effects were also observed that could not be predicted from studies of equilibrium-entangled polymers [14]. For example, during studies of polypropylene crystallization, a shift in the temperatures defining crystallization regimes was observed, so that in the partially disentangled polymer it occurred in a more orderly manner, i.e., in a lower regime, at a lower temperature [21]. Studies of mechanical properties during stretching showed that stresses increase more slowly in the strain-hardening phase if the polymer is less entangled [16]. The influence of entanglements on the generation of cavitation in a stretched polymer is significant, which also could not be inferred only from studies of equilibrium-entangled polymers. It is still not certain whether a less dense network of macromolecule entanglements affects the greater mobility of their segments and therefore lowers the glass transition temperature [18,19,22].

The list of polymers that were studied includes, among others, polylactide, polypropylene, poly(ethylene oxide), and poly(methyl methacrylate) [14]. It is only recently that research has been conducted on composites with matrices of partially disentangled polymers [23]. Barangizi et al. [24] observed in the case of a PLA composite containing 0.1–1.0 wt.% multi-walled carbon nanotubes that the disentangling of macromolecules affects the mixing of components in the melt, leading to a better dispersion of the filler.

Even fewer studies have been conducted in the case of polymer blends. Liu and Yu [25] applied high shear in the molten state to disentangle poly(ε-caprolactone) and its blend with poly(styrene-co-acrylonitrile), and then analyzed crystallization after additional controlled shear. Our group studied the properties of a polypropylene /polyethylene (PP/PE) blend when the polypropylene was partially disentangled [26]. We have shown that the process of re-entangling macromolecules occurs slowly enough that this type of polymer blends can be processed and studied. After reducing the entanglement, faster crystallization of PP was observed. The tensile experiment showed that in blends with a high PE content, the elongation at break increased with the disentanglement of PP, which was interpreted as a result of better bonding between the blend components.

The next possible step of the research on the disentangled multicomponent materials is the analysis of polymer blends, in which the miscibility of macromolecules is achieved at the nano level. Such miscibility occurs in a blend of PLA and PMMA [27]. Polylactic acid is a promising polymer as derived from renewable sources; however, its applications are limited due to poor mechanical properties [28,29] which can be modified by blending with poly(methyl methacrylate), an amorphous and transparent polymer [30]. When mixing of PLA and PMMA is carried out in the melt, miscibility is achieved at the molecular level [27,31,32,33,34], what was concluded from observing a single glass transition temperature [35].

Studies on the properties of entangled PLA/PMMA blends have focused on melt rheology [36,37,38], dynamic mechanical properties [35,36,39], tensile strength [36,40], and impact resistance [40]. Various aspects of crystallization were also examined. The course of crystallization in blends is influenced, among other things, by two factors: the composition of the blend and the dispersion of the minority component. It has been found that the presence of PMMA inhibits the crystallization of PLA. Increasing the PMMA content leads to a decrease in the equilibrium melting temperature [27,33]. The addition of PMMA reduces the cold crystallization of PLA [41]. When a growth of PLA spherulites was possible, their growth rate and the overall isothermal crystallization rate decreased with increasing PMMA content [42]. Some studies on crystallization also focused on the formation of stereocomplex crystals when PLA was blended with PMMA [31,43].

It is known that crystallization depends at the molecular level on the mobility of macromolecules, which in turn is limited by their entanglements. Hao et al. [37] used the results of a rheological frequency test to determine the equilibrium entanglement density, *ρ_e_*, in PMMA, PLA, and their blends with different compositions. Since *ρ_e_* was highest for a blend with an intermediate composition, the authors concluded that different macromolecular chains are more likely to entangle with each other than similar chains.

At least two ways are possible to obtain a PLA/PMMA blend with reduced macromolecular entanglements. The first is to dissolve and mix both polymers in a common solvent, such as methylene chloride. Research on such blends was the subject of a separate article [44]. The second approach is to disentangle one of the polymers, i.e., PLA capable of crystallization, and then mix it in the molten state in an extruder with the equilibrium-entangled PMMA. The materials obtained this way and their properties are the subject of this article. Choosing the second path provides the opportunity to analyze whether similar phenomena are observed in PLA/PMMA and previously studied PP/PE [26], i.e., whether they are universal for different pairs of polymers. Furthermore, one can compare whether the properties of PLA/PMMA blends with the same composition, but prepared in different ways, i.e., mixed in solution and those mixed in the melt, are similar or whether differences occur. We also expanded the scope of the experiments by adding studies using the dynamic mechanical thermal analysis (DMTA) technique and performing additional analyses of the crystallization process.

## 2. Materials and Methods

### 2.1. Materials

Poly(L-lactide) Nature Works 4032D (Plymouth, MN, USA) was used as one of the components of the blends. Measurement of molecular weight by the gel permeation chromatography with a multi-angle light scattering detector (GPC-MALLS) gave the following values: M_n_ = 72,000 g/mol, M_w_ = 88,000 g/mol. In the literature, higher values of M_n_ and M_w_ are often reported for the PLA [45], which results from the method used to determine them. In our case, the molecular weights were determined using a multi-angle light scattering detector, as described in detail by Kost et al. [46] and Bojda et al. [47]. Such measurements provide absolute values of molar masses that do not require the use of a correction factor.

Poly(methyl methacrylate) Sumipex Clear from Sumitomo (Tokyo, Japan) was the second component of the blends. According to the manufacturer, its molecular weight was 100,000 g/mol and the polydispersity was 1.5.

The polylactide used to prepare the blends was disentangled in a methylene chloride solution, using a procedure similar to that described by Liu et al. [12]. The polymer concentration in the solution was 5 or 0.5% by weight. Dissolution was carried out in a glass flask using a mechanical stirrer at a temperature of 30 °C. After 2 h of mixing, the solution was poured into a large amount of liquid nitrogen. Then, ethanol was added to the frozen solution in a volume three times that of the solution, which caused the PLA to precipitate as a white powder. The solution was filtered through a Teflon sieve, and the obtained powder obtained was dried under vacuum at 40 °C for 24 h. Achieving a constant PLA mass confirmed the proper choice of the end of the drying process.

PLA with varying degrees of entanglement, resulting from different concentrations in the solution, was mixed in the molten state with equilibrium-entangled PMMA. The PLA/PMMA ratio was 90:10, 80:20, or 20:80 by weight. Since PLA is sensitive to moisture, drying was performed before processing. A laboratory mini-extruder EHP-5CS from Zamak–Mercator (Krakow, Poland) was used for mixing. This co-rotating twin-screw extruder was designed for small (5 cm^3^) batches of material. The machine’s plasticizing system was equipped with a bypass valve, which allowed the plasticized material to pass through the extruder multiple times. During blending, all heating zones had the same temperature. The components were blended for 3 min at a temperature of 180 °C with a screw rotation speed of 120 rpm. The resulting blend cooled slowly in the air after leaving the extruder.

For comparison with blends having partially disentangled macromolecular chains, several additional materials were prepared by extrusion. These were equilibrium-entangled blends of PLA and PMMA, with weight ratios of 90:10 and 80:20, formed using the original pellets. In addition, previously disentangled polylactides were extruded under the same conditions to avoid processing effects when comparing properties.

Table 1 presents a complete list of the prepared homopolymers and blends, along with their abbreviations used in the text.

### 2.2. Rheological Methods

Rheological properties of the blends were studied using an Ares G2 rheometer (TA Instruments, New Castel, DE, USA) in a plate–plate configuration. Samples for testing had a diameter of 25 mm and a thickness of 0.5–1 mm. A frequency-sweep test was performed at 180 °C in the frequency range of 0.1–512 rad/s, with a strain of 1.5%. The values measured during the test included the storage modulus (*G′*), the loss modulus (*G″*), based on which the complex viscosity (η*) was determined. The complex viscosity is described by the equation, η* = G*/*ω*, where G* is a complex modulus, and ω is a frequency. The molecular mass between entanglements, *M_e_*, was determined according to the method proposed by Eckstein et al. [3], based on the calculation of the plateau modulus. For PLA, just like for a number of other polymers, due to equipment limitations, it is not possible to determine the plateau modulus directly from the *G′* = f(ω) dependence. The plateau modulus $G_{N}^{o}$ was determined using the equation:

(1) $$G_{N}^{o}=\frac{2}{\pi }\int_{-\infty }^{\infty }G”\;\left(\omega \right)dln\left(\omega \right)$$

Since the *G″* values were available only for part of the full-frequency range, the data from the low-frequency range up to the maximum were multiplied by two to obtain the value of $G_{N}^{o}$. Then, *M_e_* was determined using the equation:

(2) $$M_{e}=\frac{\rho RT}{G_{N}^{o}}$$

where *ρ* is the density of the polymer or blend, *R* is the gas constant, and *T* is the temperature.

Determining molecular masses based on Equations (1) and (2) is subject to measurement errors, related, among other things, to the limited range of measured frequencies, the accuracy of the measured modulus values themselves, and possible fluctuations in the density of entanglements in the tested material. It can be assumed that the measurement accuracy of *M_e_* is about 10%.

For description of the properties of blends, the equations proposed by Wu [37,48] can be used to determine the molecular mass between entanglements of different types of macromolecules, $M_{e12}^{o}$:

(3) $$G_{N\;blend}^{o}={\varphi_{1}}^{2}G_{N1}^{o}+{\varphi_{2}}^{2}G_{N2}^{o}+2\varphi_{1}\varphi_{2}G_{N12}^{o}$$

and

(4) $$G_{N12}^{o}=\frac{{\left(\rho_{1}\rho_{2}\right)}^{1/2}RT}{M_{e12}^{o}}$$

Here, *φ_i_* is the volume fraction of the i-th component, $G_{Ni}^{o}$ is the plateau modulus of i-th blend component, and *ρ_i_* is the density of this component. The density of the amorphous phase of PMMA is 1.19 g/cm^3^ and the density of the amorphous phase of PLA is 1.23 g/cm^3^ [49]. These values can be used as an approximation of densities of polymers in the molten state. It should be noted that $G_{N\;blend}^{o}$ as well as $G_{N1}^{o}$ and $G_{N2}^{o}$ are measured in the experiment, while $G_{N12}^{o}$ and $M_{e12}^{o}$ are calculated from the experimental data.

Thermomechanical properties, including glass transition temperatures, were studied using dynamic mechanical thermal analysis (DMTA). Tests were carried out using a DMTA Q-800 device from TA Instruments (New Castle, DE, USA) equipped with a single-cantilever clamp. Films with a thickness of 0.5–1.0 mm were tested in temperature scans from −100 °C to 150 °C at a heating rate of 2 °C/min. Experiments were conducted in a single-frequency oscillation mode at a frequency of 1 Hz with a constant strain of 0.02%.

### 2.3. Morphology Observations

The morphologies of blends were observed using a JSM 6010LA (Jeol Ltd., Tokyo, Japan) scanning electron microscope (SEM). Samples were coated with gold by sputtering, and some of them were previously fractured in liquid nitrogen to reveal the internal structure.

### 2.4. Crystallization Studies

The crystallization of blends was examined using a DSC Q-20 differential scanning calorimeter (TA Instruments, New Castle, DE, USA). In the non-isothermal experiment, the sample was heated from 0 °C to 190 °C at a rate of 10 °C/min, then cooled to 0 °C at the same rate, and then heated again to 190 °C. In the isothermal studies, the blend was melted for 2 min at 190 °C, and then the temperature was lowered to the crystallization temperature: 125 °C or 120 °C. The experiment was finished when the heat flow was steady. During the calculations of the heat of crystallization, the PLA content in the total sample mass was taken into account. In the calculations, it was assumed that the heat of crystallization in 100% crystalline PLA is 146 J/g [44].

For the detection and analysis of the periodic crystalline structure, a small-angle X-ray scattering (SAXS) technique was used. The GeniX (Xenocs, Grenoble, France) X-ray source operating at 50 kV and 1 mA was coupled to a Kiessig-type SAXS camera of 1.2 m in length. The scattered radiation was recorded using a Pilatus 100 K detector and analyzed using Image J 3.0. (National Institutes of Health, USA) software. The long period of the structure and the thickness of the PLA crystals were calculated using a correlation function approach. The correlation function shows the periodicity of the system consisting of lamellae and the amorphous phase and allows the calculation of the thicknesses of both phases.

A Linkam TAHMS 600 (Linkam Scientific, Salfords, UK) heating stage was used to observe the formation of spherulites under isothermal crystallization conditions. Because the heating stage was placed in a polarizing microscope equipped with a camera and connected to a computer, it was possible to observe and record spherulite growth. The blend samples were in the form of thin films, 10 µm thick. They were melted by raising the temperature to 190 °C, and after 3 min, the temperature was lowered at a rate of 10 °C/min to the selected crystallization temperature. The crystallization temperatures at which spherulite growth was observed were in the range of 125–158 °C.

## 3. Results and Discussion

### 3.1. Characterization of Partially Disentangled PLA Used for Blend Preparation

**Figure 1:**
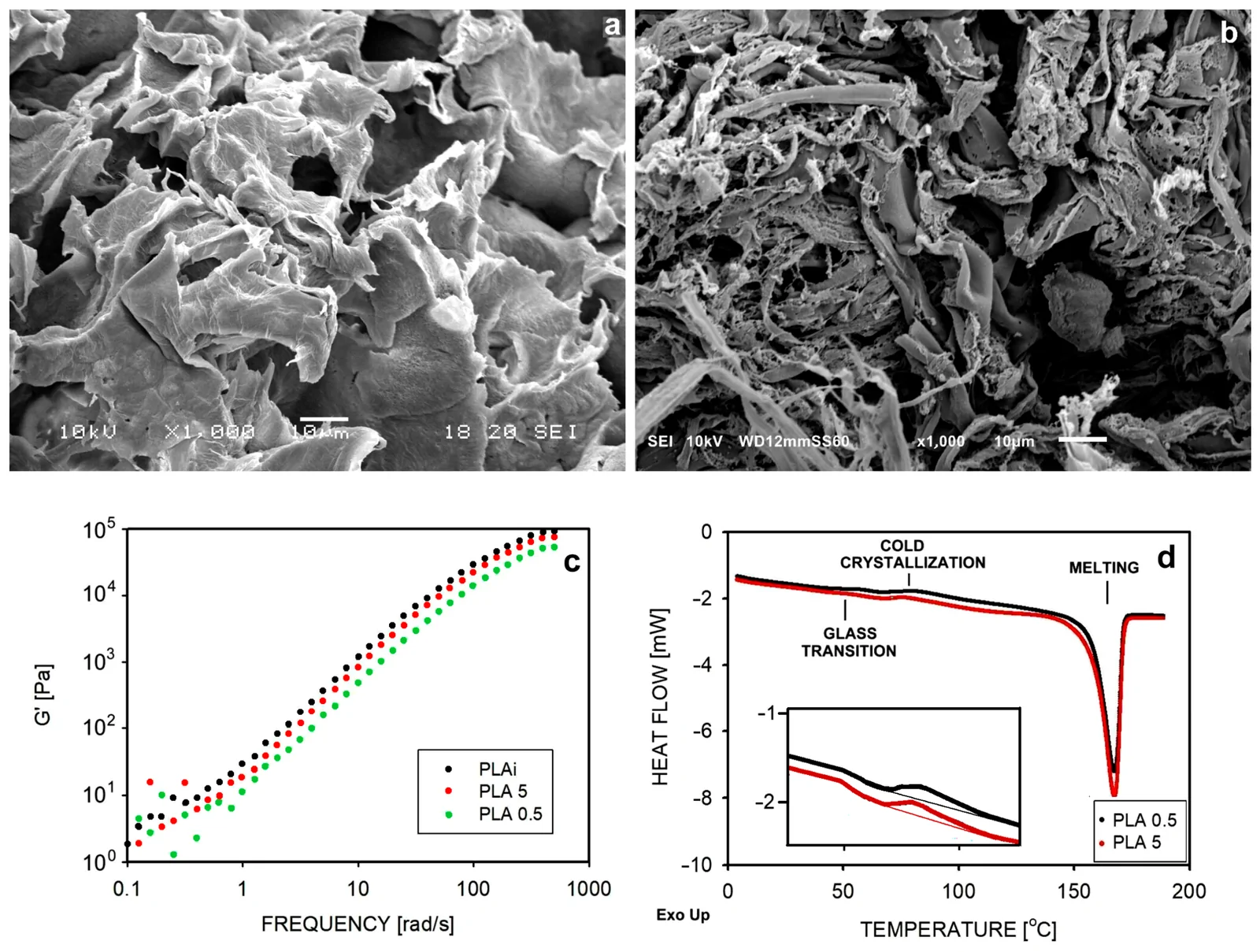
Morphology and properties of partially disentangled PLAs. The appearance of PLA-5 and PLA-0.5 powders, observed using an electron scanning microscope, is shown in (**a**) and (**b**), respectively. The dependence of the storage modulus on the frequency determined in rheological studies is shown in (**c**). (**d**) shows DSC thermograms recorded during the heating of PLA-5 and PLA-0.5 powders with a rate of 10 °C/min. The insertion contains an enlarged glass transition region and a cold crystallization maximum.

Since obtaining partially disentangled PLA and its properties have already been described several times [24,44], only information useful for the later discussion of the properties of blends is presented below. PLA after the disentangling process was obtained in the form of a white powder. The morphologies of partially disentangled powders on a micro scale are shown in Figure 1a,b. The structure of PLA-5 in the SEM images is more compact, while the less entangled PLA-0.5 has a more developed structure.

The image of PLA-0.5 shows small amounts of fibers, which is characteristic of more disentangled polymers [50]. In the rheological studies, partially disentangled PLAs were compared with fully entangled PLA-i (Figure 1c). The storage modulus *G′* depends on the entanglements in the polymer melt; therefore, the highest values obtained for PLA-i and the lowest for PLA-0.5 confirm the obtaining of materials with reduced macromolecular entanglement concentrations. The slight dispersion of the results visible in Figure 1c at low frequencies was due to the stabilization of the temperature at the beginning of the experiment. To avoid the risk of re-entanglement, the measurements were conducted immediately after the temperature was reached.

A parameter characterizing the entangled state is the molecular weight between entanglements, *M_e_*. Previously working with the same PLAs, we found that *M_e_* was 7500 g/mol for PLA-i, 8800 g/mol for PLA-5 and 14,800 g/mol for PLA-0.5 [44], which statistically corresponds to 11.7, 10.0 and 6.0 entanglements per macromolecule, respectively. In these calculations, *M_e_* was compared with *M_w_*.

The DSC curves of less (PLA-0.5) and more (PLA5) entangled polylactides heated at a constant rate were similar (Figure 1d). The glass transition (*T_g_*) was observed at 50 °C for PLA-5 and 52 °C for PLA-0.5. These values are slightly lower than the temperatures of 55–60 °C measured for fully entangled PLA. Above the glass transition, at 68 °C, cold crystallization began, with a maximum value of heat at 78 °C for PLA-5 and 82 °C for PLA-0.5. The heat of cold crystallization was very low, amounting to 1.4 J/g and 2.3 J/g, respectively. The melting occurred similarly in both PLAs, at temperatures above 110 °C, with a peak maximum at 167.6 °C. The melting heat was 46 J/g for PLA-5 and 39 J/g for PLA-0.5, which means that after taking into account the contribution of cold crystallization, the degree of crystallinity of the polymers after the process of consideration was 30% for PLA-5 and 25% for PLA-0.5.

### 3.2. Morphologies of Blends

**Figure 2:**
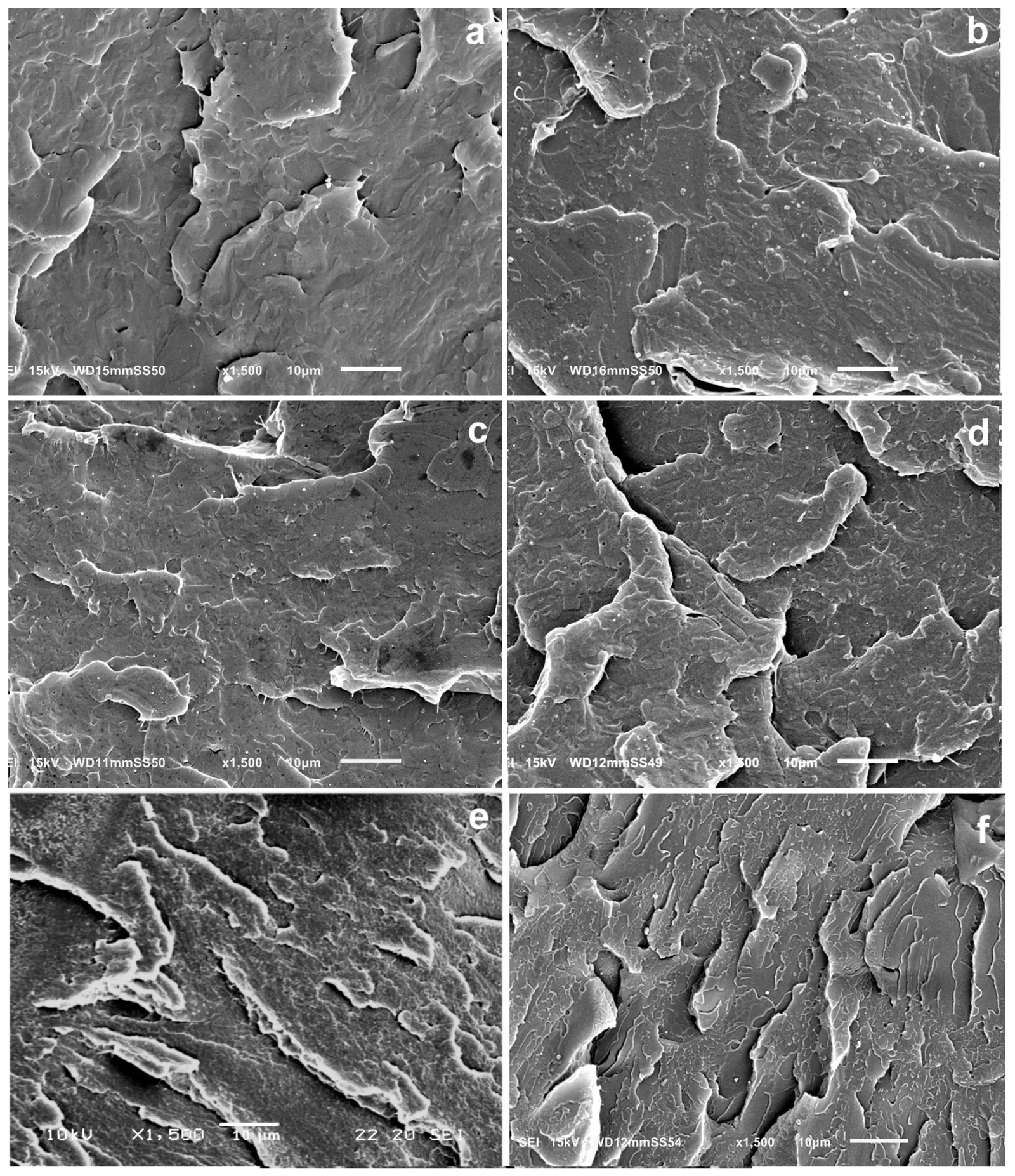
Morphologies of blends: (**a**) B-i-10, (**b**) B-i-20, (**c**) B-5-10, (**d**) B-0.5-20, (**e**) B-0.5-80, (**f**) B-i-80. The magnification in the photos is 1500 times. The white bar represents 10 μm.

According to the literature [31,32,33,34], PLA/PMMA blends obtained by melt extrusion are characterized by very good dispersion of the minority component, although some authors also observed that a small amount of the minority polymer remains undispersed [43,51]. To confirm the achievement of miscibility, morphologies of our blends were examined using SEM. Analysis of the representative images (Figure 2) showed that most of the PMMA was molecularly dispersed in PLA. Only in some blends a small number of spherical inclusions with sizes below 1 µm were visible. The presence of inclusions was observed especially in the B-i-20 blend (Figure 2b). Comparison with the B-i-10 blend shows that with increasing PMMA content, difficulties arise with its complete dispersion when the PLA macromolecules are fully entangled. In turn, the comparison of B-i-20 with B-0.5-20 (Figure 2d) shows that the disentanglement of the matrix supports the dispersion of the minority component. Figure 2e,f shows the morphologies of the blends in which the matrix was PMMA and the dispersed phase was PLA. Good dispersion of the minority component was achieved in both blends. Only single particles were visible in the images, and fewer were visible when the PLA macromolecules were less entangled.

Comparing the currently analyzed blends with those previously prepared by mixing in solution and containing up to 20% PMMA [44], it can be stated that the dispersion of PMMA has only slightly deteriorated as a result of the change in the blending method.

### 3.3. Rheological Properties in the Melt

**Figure 3:**
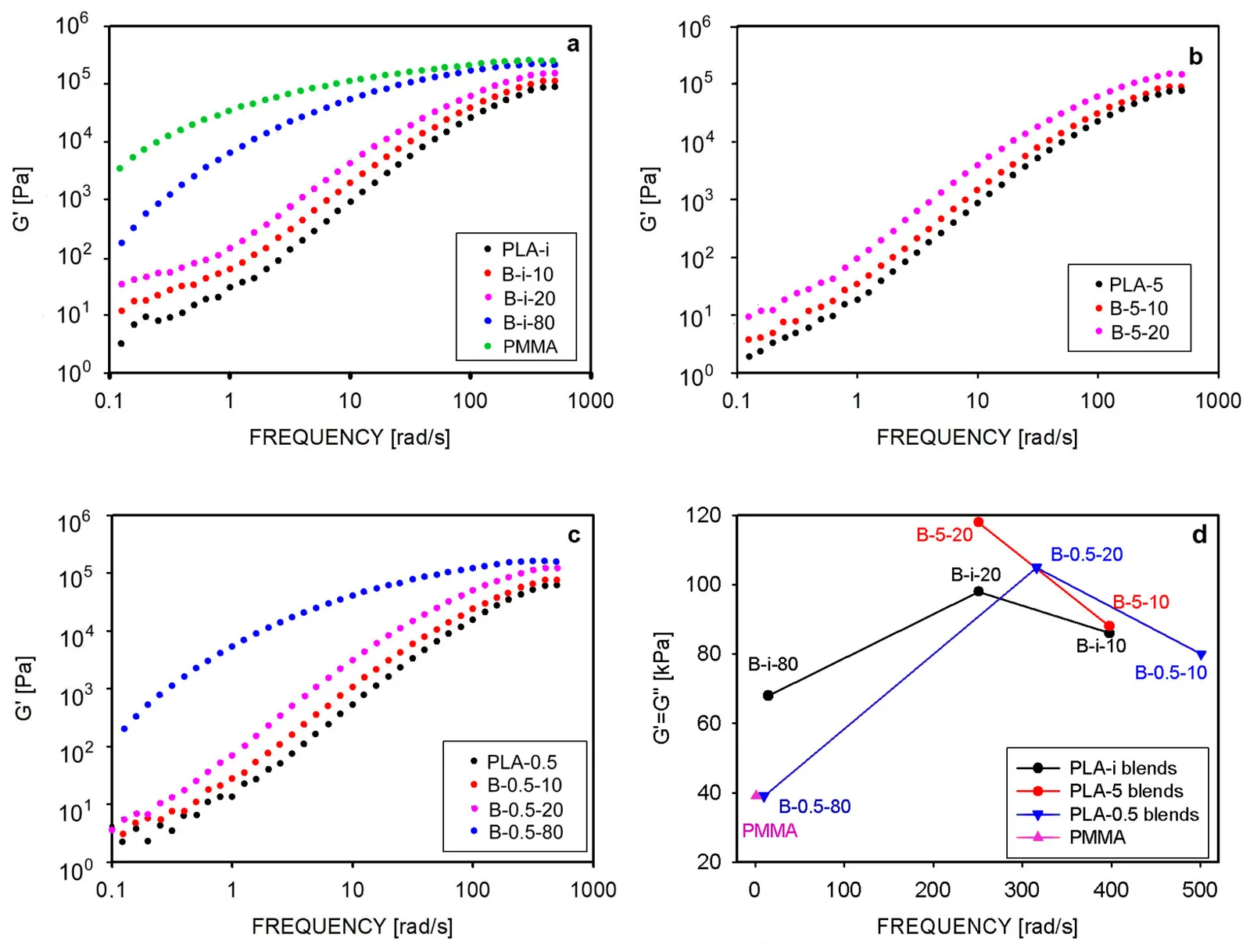
Storage modulus as a function of frequency, measured at 180 °C for homopolymers and blends (**a**–**c**). (**d**) shows the intersection points of the frequency dependence of G′ and G″.

**Figure 4:**
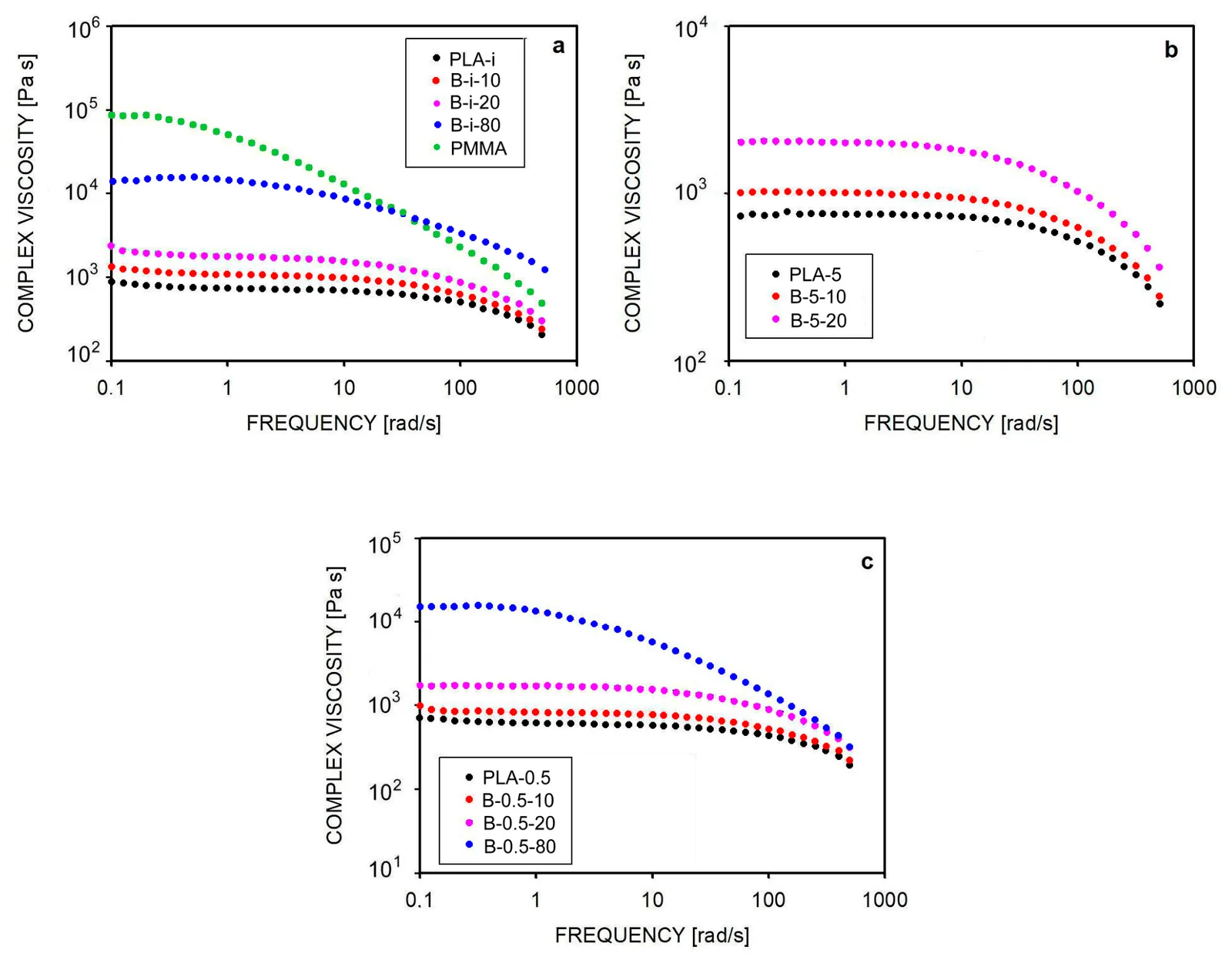
Complex viscosity of homopolymers and blends based on PLA-i (**a**), PLA-5 (**b**) and PLA-0.5 (**c**).

The rheological properties of the blends in the melt were analyzed in a frequency-sweep test. The values of the storage modulus, *G′*, and the complex viscosity, η*, as a function of frequency, are shown in Figure 3 and Figure 4 and detailed values are included in Table 2. In blends with the same level of macromolecular entanglement, it was observed that an increase in PMMA content led to higher values of *G′* and η*. As seen in Figure 3, PMMA has a higher *G′* modulus value than PLA, resulting in intermediate *G′* values measured for the blends.

Values of the *G′* modulus of the blends can be compared in the range of high frequencies, e.g., at 500 rad/s. In the case of entangled PLA-i and its blends, the modulus increased from 80 kPa to 240 kPa with increasing PMMA content from 0 to 80 wt.%. The modulus of PMMA was 250 kPa. When PLA-5 was used to prepare the blends, *G′* increased from 70 kPa for PLA-5 to 150 kPa for the B-5-20 blend. For less entangled PLA-0.5, the *G′* modulus was 60 kPa, while the B-0.5-80 blend containing 80% PMMA had a modulus increased to 160 kPa. The conclusion from the measurements of the *G′* modulus was that while its value increases with the PMMA content in the blend, the disentangling of PLA macromolecules leads to the opposite effect, i.e., a decrease in the modulus value.

One way to characterize the properties of blends is to specify the intersection point of the dependencies *G′* = *f*(*ω*) and *G″* = *f*(*ω*). The crossover frequency is inversely proportional to the relaxation time. The determined crossover points are shown in Figure 3d. There are no points for homopolymers, except PMMA, because the curves representing them did not intersect before the end of the measuring range. For all types of blends, there is a noticeable trend of the intersection point shifting toward lower frequencies as the PMMA content increases. The rheological curves for blends based on PLA-0.5 and containing up to 20% PMMA intersect at higher frequencies than their counterparts, which may indicate an effect related to the lower entanglement of macromolecules, becoming noticeable when the entanglement density is significantly reduced and thus not visible for blends based on PLA-5, compared to PLA-i.

Very similar conclusions can be drawn from the analysis of the complex viscosity measurement results, shown in Figure 4. The complex viscosity η* depended on the composition of the blend and the degree of entanglement of the macromolecules. A comparison of the values can be carried out at low frequencies (0.1 rad/s), where the viscosity plateau is close to being reached.

For PLA-i-based materials, the following results were obtained: 0.9 kPa∙s for PLA-i, 1.4 kPa∙s for B-i-10, 2.5 kPa∙s for B-i-20 and 21.0 kPa∙s for B-i-80 was. This increase was due to the higher content of PMMA, which was the polymer characterized by the highest viscosity among studied materials (88.0 kPa∙s). It is worth noting that the observed increases in η* as a function of PMMA content are lower than would be expected based on the blend component ratios. This is particularly evident in the case of the B-i-80 blend. The measured viscosity values indicate a positive effect of PLA macromolecular dispersion on the flowability of blends.

The same tendency of increasing the complex viscosity with increasing PMMA content in the blend was observed for blends based on PLA-5 and PLA-0.5. In these cases, the complex viscosities of the above homopolymers were lower than for PLA-i, which resulted from partial disentanglement of macromolecules. For partially disentangled blends with 20% PMMA content, the η* values were 2.0 kPa∙s and 1.7 kPa∙s, respectively. The viscosity of B-0.5-80 was 16.5 kPa∙s, i.e., lower than that measured for B-i-80 blend. The obtained results indicate that the disentangling of macromolecules affects the rheological properties of the blends, but the effect is not very strong.

**Figure 5:**
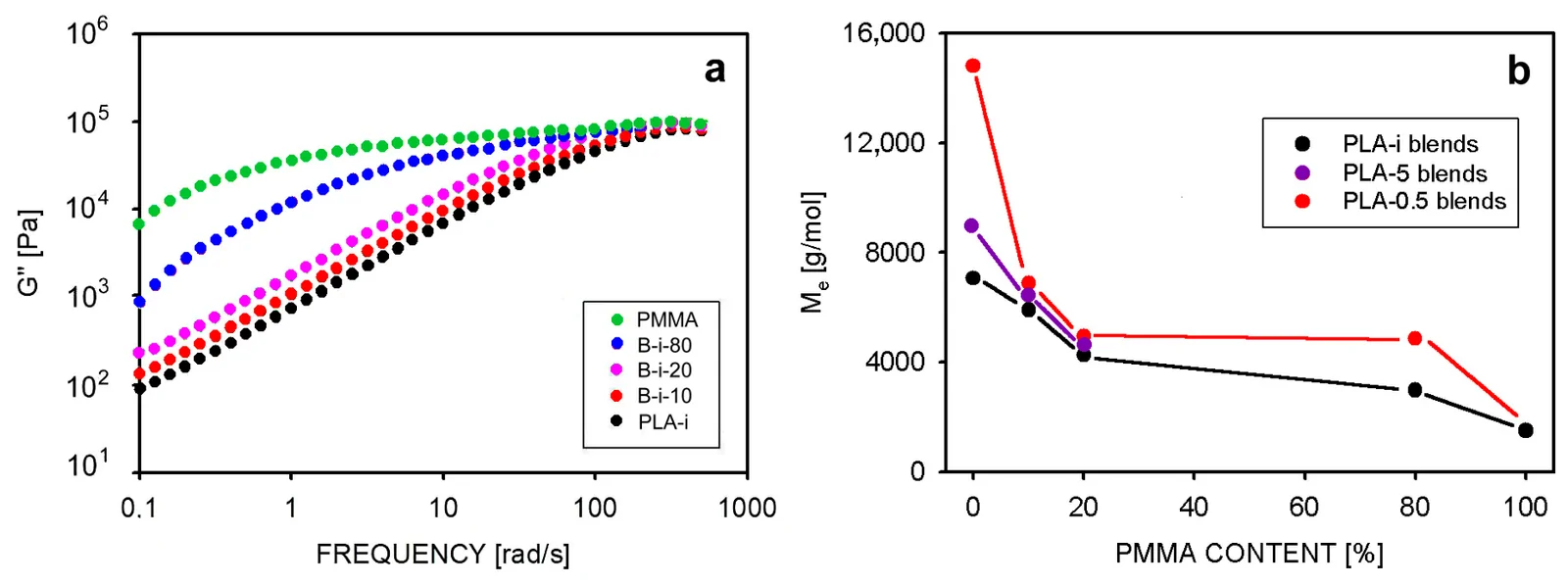
(**a**) The loss modulus as a function of frequency for PMMA, PLA-i and PLA-i-based blends; (**b**) results of calculations *M_e_* in function of PMMA content. The lines connecting the points are only placed for better visualization of the results.

Based on rheological measurements, $G_{N}^{o}$ and *M_e_* can be determined using Equations 1 and 2. The dependencies of the *G″* on frequency used in the calculations for PLA-i blends are shown in Figure 5a. As seen, over a wide frequency range, the *G″* modulus increases with the PMMA content in the blend. The results of *M_e_* calculations for PLA-i, PLA-0.5, PMMA and their blends are shown in Figure 5b. The figure does not show the results for blends with PLA-5, because among the tested materials there was no blend of this type with a high PMMA content. The *M_e_* masses for PLA-5 blends with lower PMMA content, i.e., 0–20%, had intermediate values compared to the masses measured for PLA-i and PLA-0.5 blends.

The dependencies in Figure 5b are nonlinear, both for equilibrium-entangled blends and for blends prepared using disentangled PLA. In both cases, a significant decrease in M_e_ is observed in blends with 10–20% PMMA compared to the initial PLA. This means that there is a reduction in the average distance between entanglements in the blend, greater than would be expected from the contributions of both polymers. A further increase in the PMMA content led to much smaller changes in the density of macromolecular entanglements.

Based on Equations (3) and (4), the molecular mass between entanglements of different macromolecules was calculated for the selected blends. The $M_{e12}^{o}$ mass of the B-i-20 blend was 4400 g/mol. In the case of B-0.5-20 blend, similar calculations gave a value of 4000 g/mol. These values are lower than those resulting from the proportional contribution of PLA and PMMA entanglements, as well as the results measured for the blend as a whole (4500 and 5000 g/mol, respectively), which means that macromolecules of different polymers, when present together, entangle more easily than macromolecules of a single polymer [37].

### 3.4. Rheological Properties of Blends in DMTA Studies

**Figure 6:**
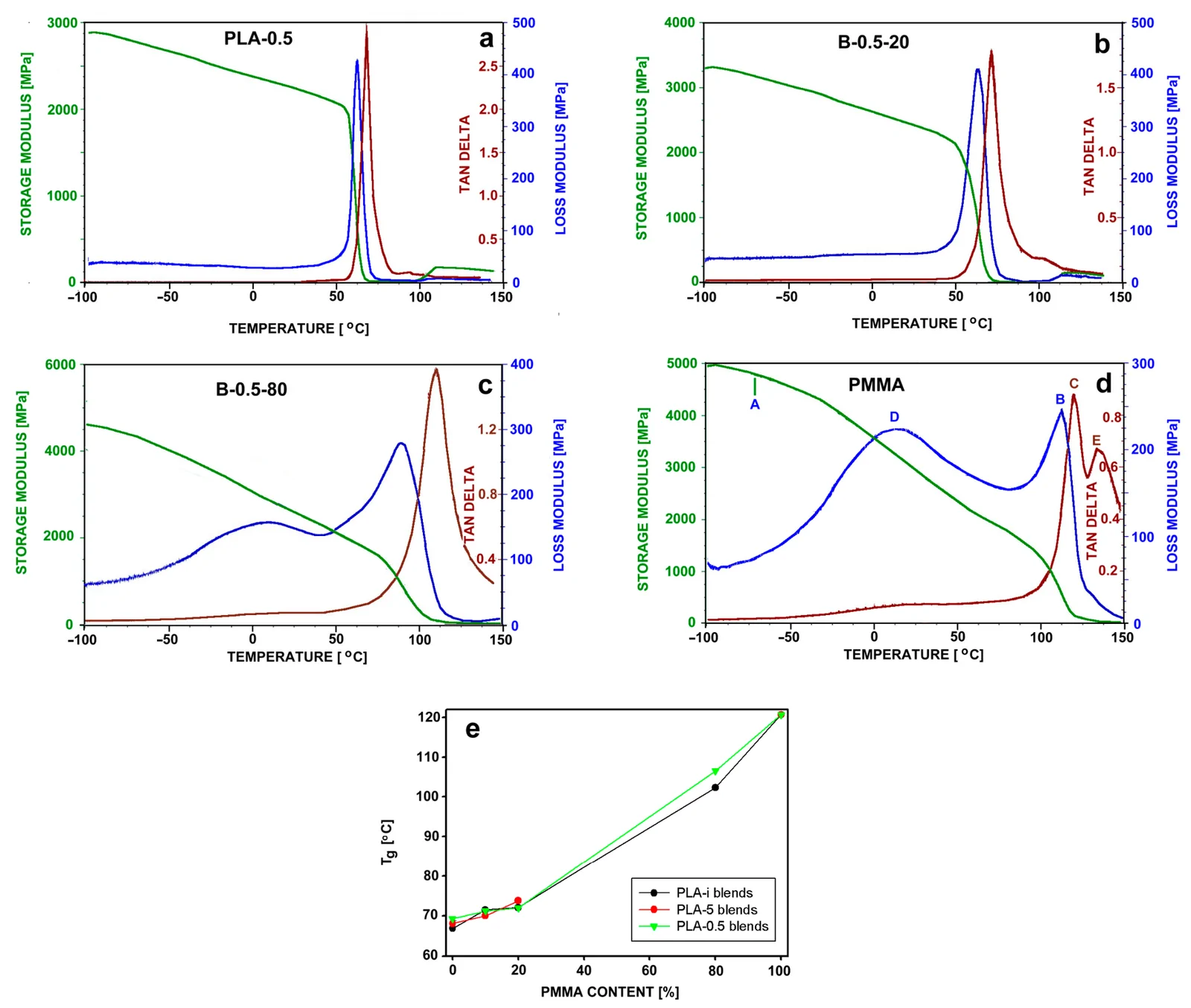
Results of measurements of the loss modulus, storage modulus, and tangent δ as a function of temperature, shown for representative materials: (**a**) PLA-0.5, (**b**) B-0.5-20, (**c**) B-0.5-80, (**d**) PMMA. In (**d**), characteristic points (A–E) are also shown, for which the measured values are given in Table 3. The dependence of the glass transition temperature, determined from the maximum of the *tan δ* curve, as a function of PMMA content in the blend, is shown in (**e**).

The viscoelastic properties of the blends were also studied using the dynamic mechanical test method, and the obtained values of storage moduli (*E′*), loss moduli (*E″*) and *tan δ* are presented in Table 3 and Figure 6. The figures show examples of changes with temperature, which are also representative for other materials. For blends containing up to 20% PMMA, the storage moduli decreased approximately linearly in the range from −100 °C to 50 °C, followed by a rapid decline. For PMMA and blends containing 80% of it, three ranges of variation in modulus *E′* with temperature can be distinguished. From −100 °C to −50 °C, it decreased slowly, then more rapidly, approximately linearly to approximately 70–100 °C, and finally, above 70–100 °C, it decreased rapidly to low values. Comparison of the *E′* value at −70 °C shows that it increases with the PMMA content. In the case of the B-i-80 blend, the measured modulus value even exceeded the modulus for PMMA.

The *E′* modulus decreases with reduction in macromolecular entanglement in the case of PLA homopolymers. In blends, this trend is maintained across the entire composition range when comparing blends of PLA-i and PLA-0.5. This can be interpreted as an effect related to the greater flexibility of less entangled macromolecules. However, the moduli of blends based on PLA-5 deviate from this pattern and are similar to those of blends with PLA-i, and the reason of this is not clear.

The curves describing the dependence of the loss modulus on temperature show one maximum in the case of blends with PMMA content of 0–20% and two maxima when PMMA dominates in the blend. Polylactides showed higher values of loss modulus at the temperature corresponding to the main maximum on the curve (at point B, see Figure 6d) than PMMA. In the case of blends with a change in composition, the *E″* moduli first increased above the value for PLA, and then at the content of 80%, they were already at a level close to the modulus of PMMA.

The temperature corresponding to the maximum on the curve (point B) is sometimes interpreted as the actual glass transition temperature. It increases from 61 to 62 °C for PLA homopolymers to 113 °C for PMMA. The increase in *T_g_* with PMMA content up to 20% is relatively small, by about 2–3 °C. Blends containing 80% PMMA have temperatures in the range of 87–89 °C, i.e., still significantly below that recorded for PMMA. A similar nature of *T_g_* changes in PLA/PMMA blends was also observed by Cousins et al. [29,33]. In homopolymers with limited entanglements, a change in the glass transition temperature with the degree of macromolecule entanglement is usually not observed [14]. The results in Table 3 show that the same is true for blends with one disentangled component. On the loss modulus curve of PMMA and blend with 80% PMMA, there is an additional broad peak, with a maximum (point D) at a temperature of 10–12 °C.

The dependence of *tan δ* on the temperature is often used to identify the glass transition temperature. The temperatures at which the *tan δ* maximum occurs are collected in Table 3, and their variability is additionally shown in Figure 6e. Similarly to analyzing changes in *E″*, one can observe an increase in *T_g_* with the PMMA content. However, the experimental points are below what would be expected from the proportion of components. This means that PLA macromolecules interact beneficially with the stiffer PMMA macromolecules, lowering the glass transition temperature.

### 3.5. Non-Isothermal Crystallziation

**Figure 7:**
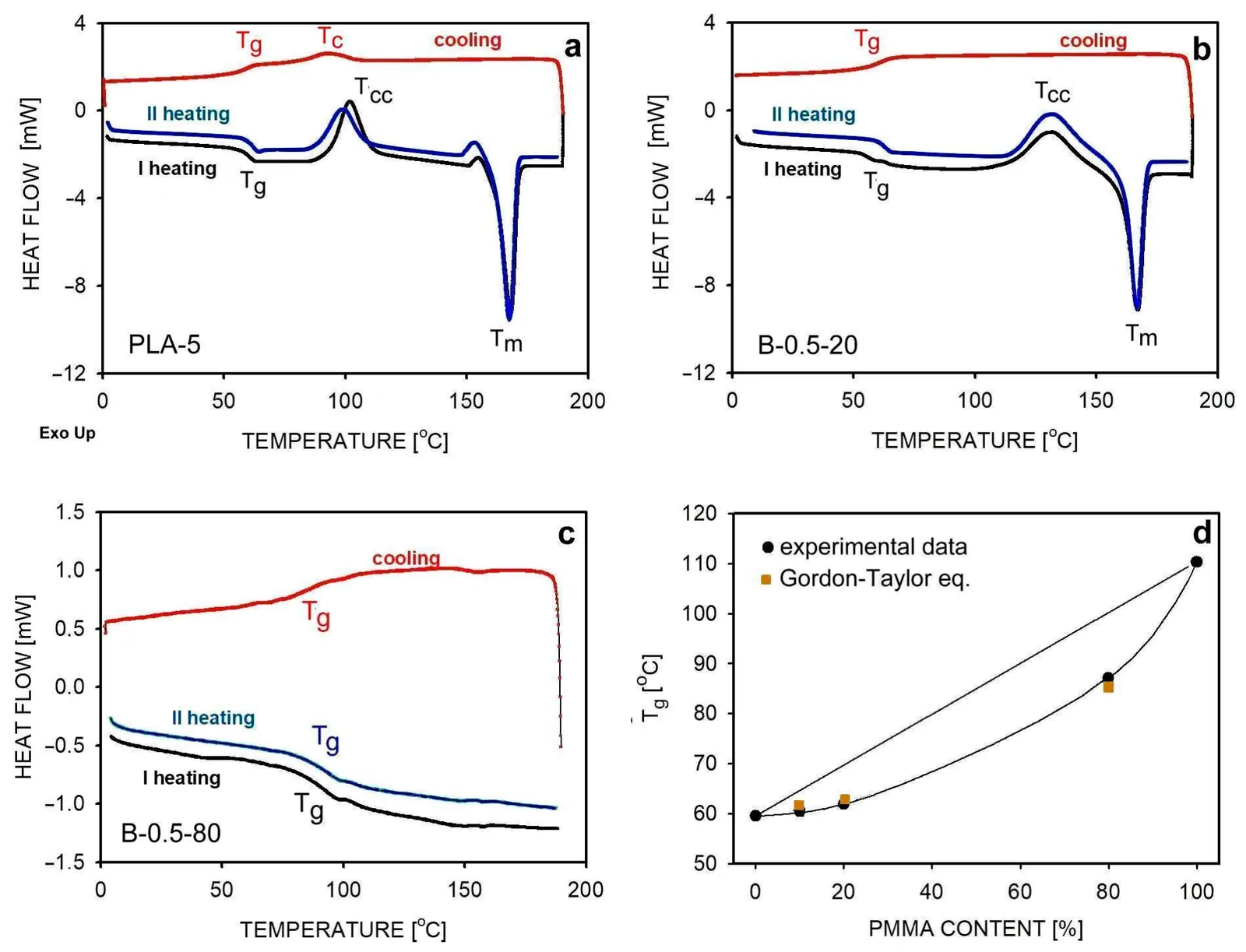
Example DSC thermograms, representative of the tested materials: (**a**) PLA-5, (**b**) B-0.5-20 and (**c**) B-0.5-80. (**d**) shows the changes in glass transition temperature for blends made from PLA-0.5, depending on the PMMA content. Temperatures were determined during cooling of the samples.

Many observations show that polymers with limited entanglements crystallize more easily [12,14,21]. To determine whether PLA and PMMA blends formed with use of one partially disentangled component behave similarly, crystallization studies were conducted under non-isothermal and isothermal conditions. The temperatures and heats of transition under non-isothermal conditions are presented in Table 4, and representative DSC thermograms are shown in Figure 7.

During the first heating in PLA and most of the blends, a *T_g_* transition was observed around 60 °C, followed by cold crystallization of PLA above 100 °C, and then PLA melting with a peak around 167 °C (Figure 7a,b). The exceptions were the B-i-80 and B-0.5-80 blends, where the PLA content was too low for effective crystallization initiation, and as a result, melting was not observed either (Figure 7c). In these blends, the glass transition temperature increased to 87–90 °C due to the dominant share of PMMA, which caused a shift in *T_g_* towards the *T_g_* of pure PMMA. The higher *T_g_* of the B-0.5-80 blend compared to the B-i-80 is likely the result of increased interaction of the more mobile PLA chains with PMMA chains in the B-0.5-80 blend. This is consistent with the *T_g_* observations using DMTA (Figure 6d) and with the previously discussed rheological behavior of the blends. Additionally, during the first heating in the case of PLA-5 and PLA-0.5, a small α’-α transition signal was observed at 153–154 °C. It should be noted that the effects observed during the first heating depend on the uncontrolled thermal history of the extruded material, while the effects observed during cooling and second heating depend on the material structure and the controlled experimental conditions.

The second step of the DSC measurements was cooling, during which, for most materials, only the glass transition was visible. Figure 7d shows changes in the glass transition temperature for blends made from PLA-0.5, depending on the PMMA content. It is clearly visible that this relationship is not linear, but can be described by the Gordon–Taylor equation [52]:

(5) $$T_{g}=T_{g1}+kw_{2}\frac{T_{g2}-T_{g1}}{w_{1}+kw_{2}}$$

where *w_i_* is the weight fraction of the i-th component, *T_gi_* is the glass transition temperature of the i-th component, and *k* is the fitting parameter. The *k* value is a miscibility measure, representing the interaction between two polymers. The data from Figure 7d can be best fitted using the Gordon–Taylor relationship if k = 0.27. A low *k* value, i.e., below one, indicates a weak interaction between the two polymers [41]. As expected, the dependence of *T_g_* on the PMMA content obtained from DMTA experiments (Figure 6e) and DSC (Figure 7d) are similar.

During cooling, a weak crystallization effect (i.e., 2–3%) was observed at 91–92 °C for the disentangled PLA, but in the blends, this was suppressed by the presence of PMMA macromolecules. These macromolecules hindered the movement of PLA macromolecules to the crystal growth sites. Combined with the short time available for crystallization, restricted mobility resulted in a lack of crystallinity.

During the second heating, similar thermal phenomena were observed as during the first heating, i.e., a single glass transition, cold crystallization of PLA, and finally the melting of PLA crystals. Cold crystallization and melting were not observed in the B-i-80 and B-0.5-80 blends. Changes in the glass transition temperature of the blends were small with 0–20% PMMA content, while blends containing 80% PMMA had significantly higher temperatures.

In the blends where cold crystallization of PLA was observed, the temperature corresponding to the crystallization peak depended on the PMMA content and the degree of entanglement of the macromolecules. As the PMMA content in the blend increased, the cold crystallization occurred later. Comparing the *T_cc_* of the blend with the *T_cc_* of the corresponding homopolymer, a temperature shift of 9.8 °C was observed for the B-i-20 blend, 24.6 °C for the B-5-20 blend and 31.7 °C for the B-0.5-20 blend. The observed trend is similar to the one recorded during the first heating. This indicates that PMMA macromolecules more strongly hinder the crystallization of less entangled PLA macromolecules. A possible reason is that the better dispersion of PMMA macromolecules at the molecular level in partially disentangled blends increases their influence on the mobility of moving PLA macromolecules.

PLA crystallization usually includes two components: crystallization during cooling and cold crystallization. Their contribution for homopolymers and the studied blends was different, and sometimes it was hard to measure the crystallization heat components accurately. However, the total crystallization heat must equal the melting heat. Therefore, analysis of the heat of melting provided additional information about the crystallization process. The melting of all materials tested, which were previously able to crystallize, occurred at a similar temperature, with a maximum in the range of 165.8–168.2 °C. The heat of the melting of homopolymers ranged from 33 to 39 J/g and was higher for the less entangled PLA. The heat of melting slightly increased (3–4 J/g) with the increase in PMMA content in the blend, which may be the result of additional nucleation by heterogeneous nuclei initially present in PMMA. The behavior of the B-i-20 blend was different, with a heat of melting of only 13.9 J/g. This blend also showed a lower heat of melting compared to other blends during the first heating. It can be assumed that with such a composition and degree of macromolecule entanglement, the presence of PMMA effectively limits the mobility of PLA macromolecules, and the time required for more intense crystallization is longer than available. When the PMMA content was higher, as in the case of B-0.5-80 and B-i-80, crystallization did not occur at all.

### 3.6. Isothermal Crystallization

**Figure 8:**
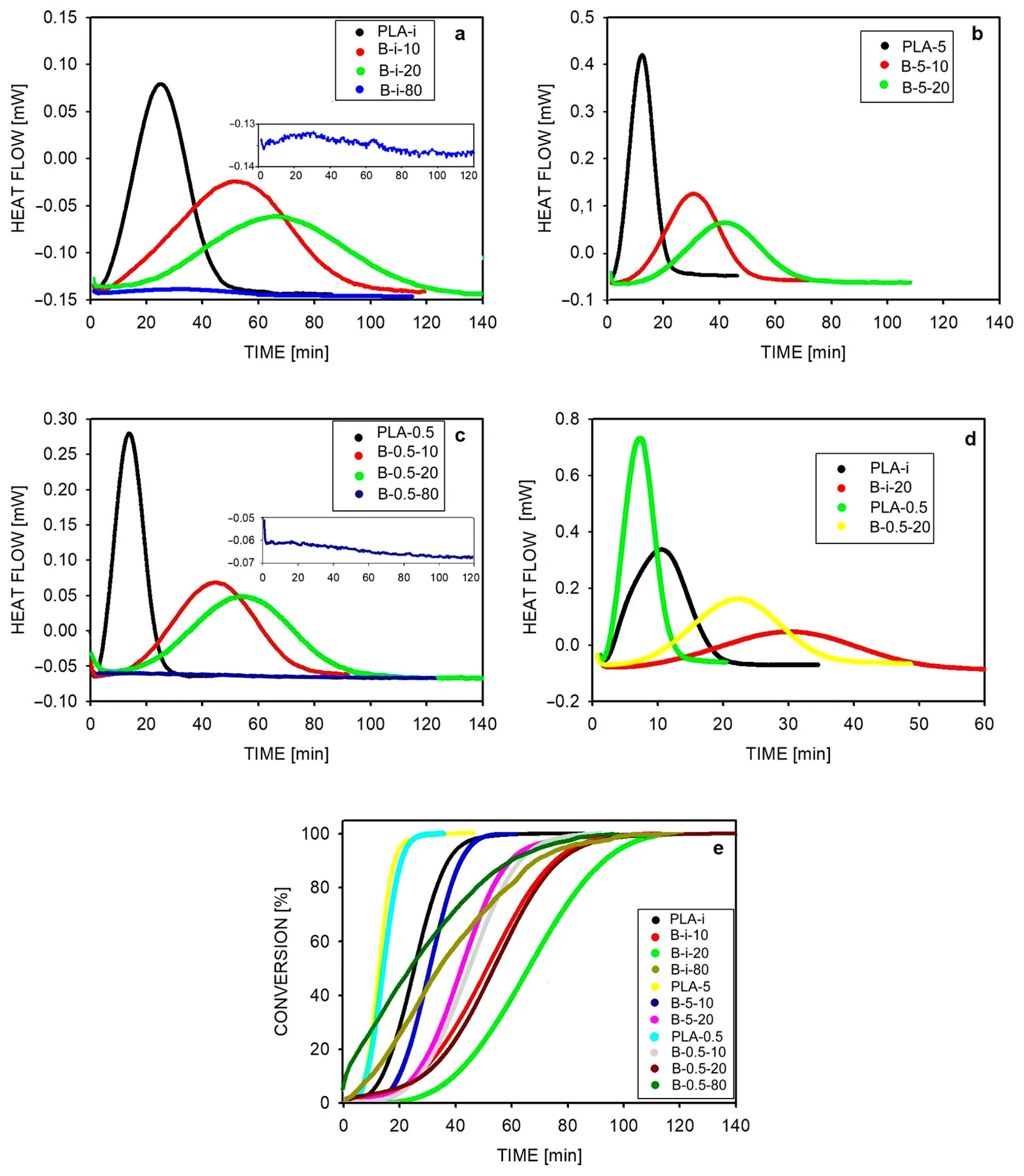
(**a**–**c**) Isothermal crystallization of homoplymers and blends at a temperature of 125 °C. Inserts in the drawings show details of the crystallization of blends containing 80% PMMA; (**d**) isothermal crystallization of homopolymers and blends at a temperature of 120 °C; (**e**) conversion of the melt to the crystalline phase for samples crystallized at 125 °C.

Since the crystallization time under typical non-isothermal conditions is short, some behaviors resulting from various levels of entanglements in blends might not have been revealed. An example could be whether crystallization occurs or not in blends with 80% PMMA. For this reason, crystallization studies were also carried out under isothermal conditions, at two crystallization temperatures: 125 °C and 120 °C. The measurement results are shown in Figure 8 and Table 5.

**Figure 9:**
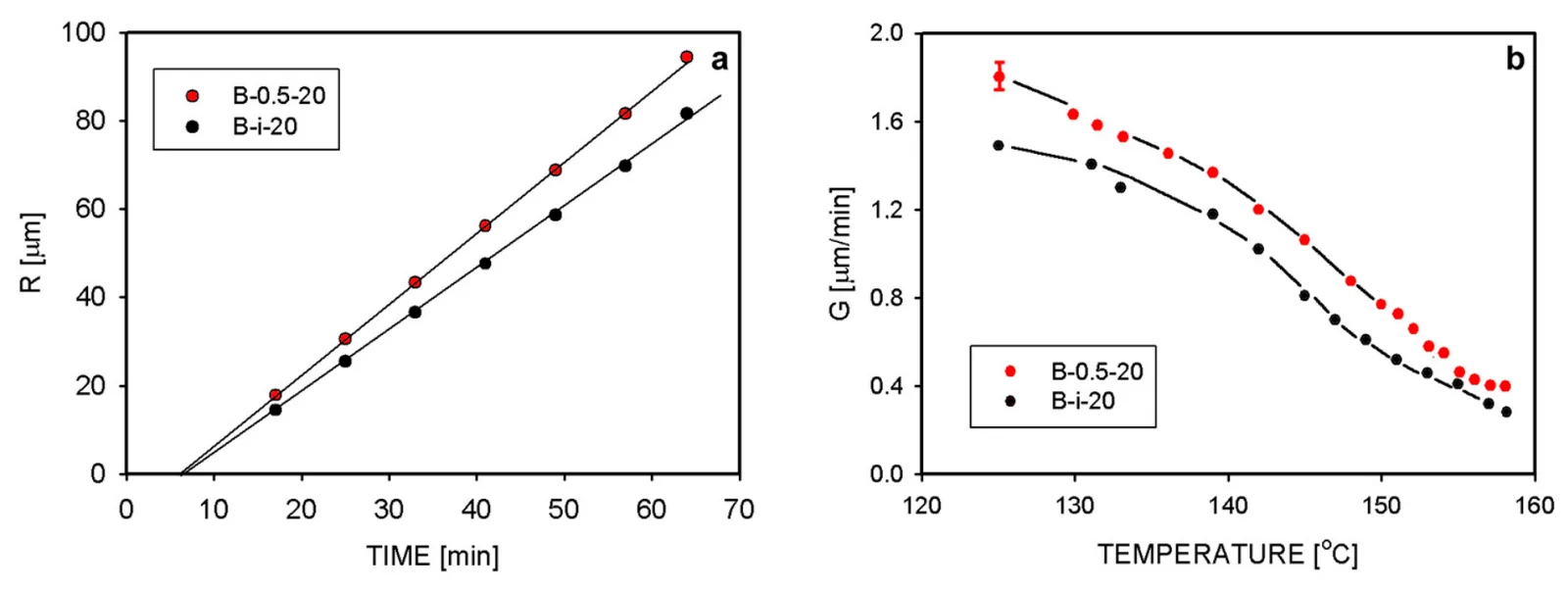
(**a**) Increase in the radius of selected spherulites with time of isothermal crystallization at 131 °C; (**b**) spherulite growth rates in B-i-20 and B-0.5-20 blends measured at different crystallization temperatures.

The first observation from the isothermal studies was that the characteristic crystallization times, i.e., the onset time, the time of maximum thermal effect and the time of completion of crystallization, increased with increasing PMMA content. This trend was independent of the entanglement of the PLA used. The second observation was that blends made from partially disentangled PLA-5 and PLA-0.5 had shorter crystallization times than blends formed from entangled PLA-i. Surprisingly, the shortest crystallization times were measured for PLA-5 blends, not for PLA-0.5 blends. The crystallization process is controlled by two factors: nucleation density and crystal growth rate. The crystal growth rate, as evidenced by the previous observations and the subsequent discussion of the results in Figure 9, is greater for disentangled polymers and blends. Thus, the unexpected crystallization times for PLA-5 blends may result from the previously observed reduction in the number of nuclei present in PLA after the disentanglement process [24], greater in the case of the less entangled PLA-0.5.

Both blends containing 80% PMMA, which did not crystallize during non-isothermal tests, showed some ability to crystallize at 125 °C. Blend B-0.5-80 crystallized slightly faster and with a higher heat of crystallization. In the B-0.5-80 blend, a degree of crystallinity of 9% was achieved, while in the equilibrium-entangled B-i-80 blend only 6%.

Figure 8d shows, for selected materials, the heat flow as a function of time at a temperature of 120 °C. Crystallization at 120 °C was faster than expected at 125 °C. Comparing the materials examined, crystallization of the blend was slower than that of the homopolymer. Disentangling shortened the process time for both homopolymers and blends.

The progress of crystallization over time can be illustrated by showing the degree of transformation of the melt into the crystalline phase. The relationships for materials tested at a temperature of 125 °C are shown in Figure 8e. A useful parameter describing the isothermal crystallization process is a half-time of conversion *t*_50%_. It is often close to, but not necessarily equal to, the time of occurrence of the maximum thermal effect. For the tested materials, differences between *t_max_* and *t*_50%_ were visible for the blend with a high PMMA content, which is consistent with the observation that the dependence for these blends does not have a symmetrical peak character, as in the case of other materials shown in Figure 8a–c. Analysis of the t_50%_ results in Table 5 leads to the conclusion that the disentangled PLA-5 and PLA-0.5 crystallized much earlier than the entangled PLA-i. The addition of PMMA to PLA retarded crystallization. However, the presence of 20% PMMA only moderately increased the time compared to 10% PMMA. Half-times for blends containing disentangled PLA macromolecules were significantly shorter than the times required for the crystallization of entangled PLA macromolecules in the blends. A characteristic feature of these studies was the prolonged crystallization half-time for the less entangled PLA-0.5 blends compared to the PLA-5-based blends. As already mentioned, this resulted from limited nucleation. In the B-i-80 and B-0.5-80 blends, crystallization started after reaching the temperature of 125 °C, and since the process was most intense in the first 20–30 min, the measured half-times were only 35 and 24 min, which is shorter than in the blends with 20% PMMA content.

Table 5 also provides information on the heat of crystallization. For homopolymers and blends containing 10 or 20% PMMA, it ranged from 40.9 to 50.3 J/g, corresponding to a crystallinity of 28–34%. This heat was higher in the blends containing more PMMA, likely due to increased nucleation. It was hard to notice the impact of the level of entanglement on the final crystallinity. The hindered movement of macromolecules when PLA was dispersed in PMMA, as in the B-0.5-80 and B-i-80 blends, resulted in a low measured heat of crystallization.

To better characterize the PLA crystals growing in the isothermal DSC experiment, the SAXS technique was used, which allowed for determining their average thickness. Scattering patterns on previously crystallized samples were analyzed using a correlation function. The crystallinity of the tested materials was assumed to be approximately 30%, so the smaller of the two obtained layer thicknesses represented the PLA crystal thickness. Measurements were not performed for the B-i-80 and B-0.5-80 blends due to their low crystallinity. Table 5 presents the long period and crystal thickness determined from the SAXS results. The long period for the materials tested ranged from 18 to 23 nm. It increased with increasing PMMA content.

The thickness of the PLA homopolymer crystals increased with the disentanglement of macromolecules from 4.7 to 6.0 nm. In PLA/PMMA blends with an entangled macromolecule, the crystal thickness remained unchanged. However, in blends containing disentangled PLA macromolecules, the crystal thickness decreased with increasing PMMA content, and this decrease was faster in blends with a less entangled structure. The measured values come both from the process of crystal formation itself and from their thickening while staying longer at the crystallization temperature. The expected better dispersion of PMMA in a less entangled blend makes it harder for PLA macromolecules to move, so the crystals end up growing smaller in the available time. The process of crystal thickening was also hindered by the presence of PMMA; the crystals grew more slowly, and even considering the longer time available for annealing, this did not lead to changes in the thickness ratio of the materials studied. The above description does not fully explain the results for PLA-i-based blends. It is possible that in the fully entangled materials, the thickening effect was small, and the thickness changes were within the measurement accuracy of ±0.2 nm. The increasing difference between the long period and crystal thickness with PMMA content confirmed that the total amount of amorphous phase in the blends increased due to the greater presence of amorphous PMMA.

### 3.7. Spherulitic Structure

Observations for PLA and its blends with PMMA have shown that a spherulitic structure is usually formed during crystallization [44]. Spherulites were observed in our blends during non-isothermal crystallization as well as in the case of isothermal crystallization carried out in a wide range of temperature, i.e., 125–158 °C. Observations were carried out in polarized light passing through a sample. Under non-isothermal conditions, a large number of spherulites formed during cold crystallization in the tested samples. Because their sizes did not exceed 5 µm, further studies on the spherulitic structure formation were conducted under isothermal conditions.

Analysis of spherulite growth at controlled temperatures, i.e., in isothermal conditions, allowed for a better characterization of the crystallization process in the studied materials [53]. The first question to investigate in blends with a single crystallizable component is whether the non-crystallizing component is trapped inside the growing spherulites or is pushed ahead of their front. In the former case, the growth rate should be constant; in the latter, it should slow down with time. The growth rate of spherulites also depends on degradation, which accelerates growth, and in the case of a disentangled polymer on the rate of a possible re-entanglement process, which slows down spherulite growth over the course of the experiment. Which of these effects applies to the tested materials was clarified by measuring the increase in the radius of the selected spherulite with time at the crystallization temperature.

The results for the B-i-20 and B-0.5-20 blends measured at 131 °C are shown in Figure 9a. A higher temperature was chosen than in the case of DSC measurements in order to limit the number of growing spherulites and thus enable their observation over a longer period of time. The straight lines describing the results indicate that in our blends the PMMA was occluded within the spherulites and that no degradation or re-entanglement occurred. From the slope of the lines, it can be calculated that the growth rate of spherulite B-0.5-20 was 1.6 µm/min and that of spherulite B-i-20 was 1.4 µm/min. In the case of radial spherulites with the crystallization of the lamellae constituting them, the spherulite growth rate is equal to the lamella growth rate.

Figure 9b shows the growth rates of spherulites in the B-i-20 and B-0.5-20 blends measured at different crystallization temperatures. As expected, the spherulite growth rate decreased with increased crystallization temperature. Furthermore, it can be seen that it was higher across the entire range for the blends containing less entangled macromolecules. This confirms previous observations that macromolecule entanglements hinder crystallization.

**Figure 10:**
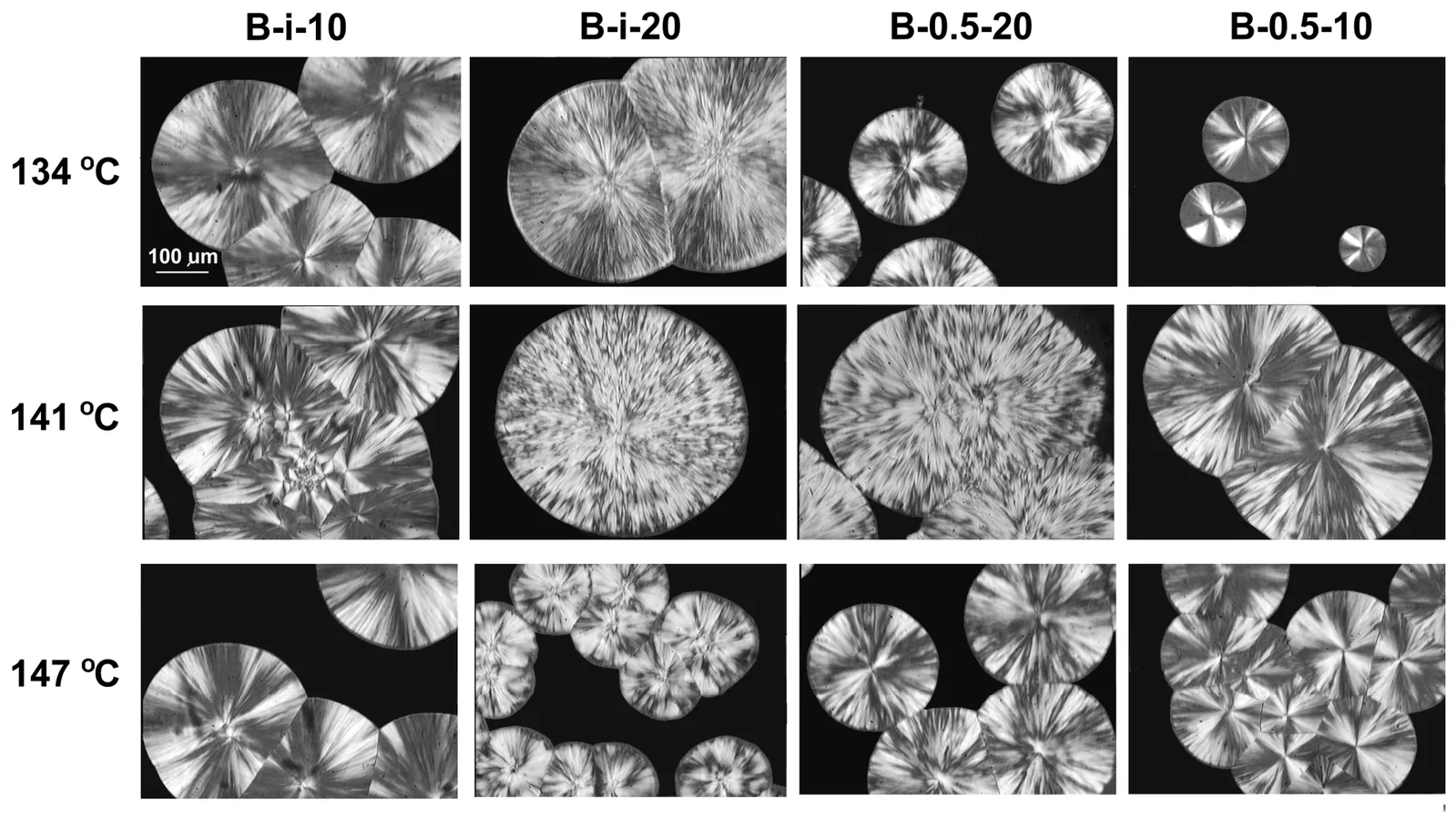
Morphologies of spherulites crystallized at selected temperatures.

One of the features characterizing the formation of PLA spherulites is that in a certain temperature range; instead of a purely radial structure, a banded structure is observed, and these rings are usually not very regular in PLA [54,55]. In banded spherulites, the growing lamellae rotate relative to the direction of illumination, which, when polarized light is used, leads to the observed dark and light rings. No studies have been conducted to date to determine whether banded spherulites occur when macromolecular entanglements are limited. Appropriate tests were carried out for blends B-i-10, B-i-20, B-0.5-10 and B-0.5-20. Examples of morphologies of spherulites are presented in Figure 10.

Radial spherulites grew in the tested blends if the isothermal crystallization temperature was below 135 °C or above 147 °C. However, in the temperature range of 135–146 °C, the structure of spherulites gradually changed, with the differences in relation to radial spherulites being the greatest at 141 °C. The spherulites visible in the photos do not have fully formed rings, and the morphology of the spherulites of the blends containing 10 and 20% PMMA differs significantly. The finer structure of the B-i-20 and B-0.5-20 spherulites is probably due to the higher content of PMMA. The evolution of the spherulite structure from radial to annular and vice versa occurs gradually, but the process seems to start about 1–2 °C earlier in blends with partially disentangled macromolecules. The relationship between the morphology of spherulites and the density of macromolecular entanglements is the subject of separate, ongoing studies.

## 4. Conclusions

The aim of this work was to determine the properties of a PLA/PMMA blend affected by limiting the entanglement of PLA macromolecules. Additionally, we investigated how the change in properties due to disentanglement relates to changes resulting from varying PMMA content in the blends. Unlike the previous work [44] in which macromolecules of both components were simultaneously disentangled during the formation of the blend-in solution, this time the reduction in entanglements in the blends was achieved by using one partially disentangled polymer and forming the blend through an extrusion process. Microscopic examination (SEM) confirmed the obtaining of homogeneous blends, with a level of component dispersion comparable to the first method.

The characterization of the properties of the produced materials began with rheological tests of the melted materials. Significant differences were observed in the moduli G′, G″ and complex viscosity η*, which were smaller in the blends prepared from less entangled polylactide. The results confirmed that not only homopolymers, but also blends prepared from them, were characterized by different degrees of macromolecular entanglement. Regardless of the PMMA content in the blend, the molecular mass between entanglements was higher for blends made using less entangled PLA. Calculations of the average mass between entanglements formed by macromolecules of different polymers, i.e., PLA and PMMA, showed that macromolecules of different polymers, when present together, entangle more easily than macromolecules of a single polymer.

The analysis of dynamic mechanical properties in the solid state mainly allowed us to determine how glass transition temperature changes with the blend composition and the degree of macromolecule entanglement. Since PMMA had a much higher glass transition temperature than PLA, the *T_g_* of the blend increased with PMMA content. However, this relationship was not linear, which meant that the PLA macromolecules interacted favorably with the stiffer PMMA macromolecules, lowering the glass transition temperature of the blend. No change in the glass transition temperature was noticed by increasing the distance between polymer entanglements. The reason is that the segments of macromolecules, whose movements are released during the glass transition, are smaller in size than the distance between entanglements. Analysis of the elastic modulus *E′* values showed that, when comparing PLA-i-based blends with PLA-05-based blends, it decreased with reduction in macromolecular entanglement, which can be attributed to the greater freedom of deformation of the macromolecular network. However, PLA-5-based blends exhibited higher *E′* values, indicating that the relationship between entanglement and elastic modulus has not been fully elucidated.

A large part of the research focused on the crystallization of PLA in the presence of non-crystallizing PMMA. First, tests were carried out under non-isothermal conditions. DSC analysis showed that differences in *T_g_* results appeared only in blends with 80% PMMA, where the dominant rigid PMMA increased the *T_g_* value from approximately 60 °C to 87–90 °C. It was also observed that the PMMA chains restricted segmental mobility more than those PLA chains that had fewer entanglements. PLA crystallization in the blend was hindered by the presence of PMMA, which was manifested by a higher cold crystallization temperature and its absence at 80% PMMA content. Blends with partially disentangled PLA cold-crystallized at lower temperatures than equilibrium-entangled blends. This demonstrated the beneficial effect of macromolecule disentanglement on the crystallization process.

The crystallization process, both under isothermal and non-isothermal conditions, was controlled by two opposing factors: the degree of macromolecular entanglement and the content of PMMA macromolecules in the blend. Since PLA macromolecules were involved in the crystallization, their greater mobility after reducing the density of entanglements promoted the whole crystallization process. However, these macromolecules, moving toward the sites of crystal formation, encountered PMMA macromolecules along the way, which acted as obstacles, delaying the crystallization process and limiting the achievable degree of crystallinity. These obstacles were particularly noticeable in blends containing 80% PMMA. If the time available for crystallization was sufficiently long, spherulites were observed forming in the blends. PMMA macromolecules were confined within the spherulite structure. No degradation effects or re-entangling of macromolecules were observed during the experiment. In a large temperature range, the growth rate of spherulites, equal to the growth rate of lamellar crystals, was higher for the less entangled blend, which resulted from the easier of movement of the macromolecular chains mentioned.

## Figures and Tables

**Table 1:** List of studied materials.

Abbreviation	Concentration of PLLA in Solution During Disentangling %	PLA/PMMA Weight Ratio in the Blend	Method of Preparation Material for Studies
Equilibrium-entangled homopolymers			
PMMA PLA-i			PMMA pellets
			PLA pellets
Partially disentangled polylactides			
PLA-5 PLA-0.5	5.0 0.5		Disentangling in solution and extrusion
Blends of entangled components			
B-i-10		90:10	Extrusion
B-i-20		80:20	
B-i-80		20:80	
Blends with one partially disentangled component			
B-5-10	5.0	90:10	Disentangling of PLA in solution, followed by extrusion with PMMA
B-5-20	5.0	80:20	
B-0.5-10	0.5	90:10	
B-0.5-20	0.5	80:20	
B-0.5-80	0.5	20:80	

**Table 2:** *G′* modulus values at a frequency of 500 rad/s, maximum *G″* modulus values, and complex viscosity η* values at a frequency of 0.1 rad/s.

Material	G′ [kPa]	G″ [kPa]	η* [kPa∙s]
PMMA	250	91	88.0
PLA-i	80	81	0.9
B-i-10	90	86	1.4
B-i-20	180	102	2.5
B-i-80	240	94	21.0
PLA-5	70	84	0.8
B-5-10	90	88	1.0
B-5-20	150	122	2.0
PLA-0.5	60	79	0.6
B-0.5-10	80	83	1.0
B-0.5-20	145	86	1.7
B-0.5-80	160	66	16.5

**Table 3:** Storage modulus (*E′*), loss modulus (*E″*) and glass transition temperature (*T_g_*) determined at characteristic points A-E (marked in Figure 6d) of the curves measured in DMTA test. For *E*′, the value measured at −70 °C was considered characteristic. *T_max_* is the temperature of the second maximum on the *E″* curve and *T_max_*_1_ is the temperature of the second maximum on the *tan δ* curve.

Material	E′ (A Point) [GPa]	E″ (B Point) [MPa]	T_g_ (B Point) [°C]	T_g_ (C Point) [°C]	T_max_ (D Point) [°C]	T_max1_ (E Point) [°C]
PMMA	4.75	245	112.7	120.7	12.0	135.1
PLA-i	3.10	424	61.7	66.9		
B-i-10	3.50	495	63.8	71.5		
B-i-20	3.80	491	63.2	72.1		
B-i-80	5.16	315	87.1	102.3	11.1	120.0
PLA-5	2.87	443	62.1	68.1		
B-5-10	3.80	521	62.6	70.0		
B-5-20	4.39	543	64.0	73.8		
PLA-0.5	2.78	280	61.3	69.3		
B-0.5-10	2.89	380	64.6	71.2		
B-0.5-20	3.18	409	64.7	72.0		
B-0.5-80	4.40	279	89.0	106.5	9.8	

**Table 4:** Results of the non-isothermal crystallization studies using DSC. *T_g_*–glass transition temperature, *T_cc_*—temperature of cold crystallization, *H_cc_* –heat of cold crystallization, *T_m_*—melting temperature, *H_m_*—heat of melting, *T_c_*—crystallization temperature, *H_c_*—heat of crystallization.

Material	First Heating					Cooling			Second Heating				
	T_g_ [°C]	T_cc_ [°C]	H_cc_ [J/g]	T_m_ [°C]	H_m_ [J/g]	T_g_ [°C]	T_c_ [°C]	H_c_ [J/g]	T_g_ [°C]	T_cc_ [°C]	H_cc_ [J/g]	T_m_ [°C]	H_m_ [J/g]
PLA-i	62.2	131.1	32.9	167.9	33.5	60.3			62.9	132.9	32.2	167.1	33.3
B-i-10	61.5	129.8	35.2	167.1	37.7	62.1			62.6	133.9	36.3	166.7	37.2
B-i-20	61.9	140.6	27.5	167.1	30.1	61.1			63.3	142.7	12.1	166.8	13.9
B-i-80	87.4					81.6			86.4				
PLA-5	60.6	102.0	34.9	168.3	36.5	60.2	92.3	4.9	62.0	99.5	23.3	167.6	38.0
B-5-10	61.8	116.2	37.1	168.2	40.7	60.6			62.6	115.3	38.4	168.2	39.5
B-5-20	62.8	125.3	40.9	165.9	41.8	60.3			63.2	124.1	41.1	165.8	42.9
PLA-0.5	61.1	101.5	32.7	167.7	38.8	59.5	91.0	2.4	62.0	101.7	30.6	167.3	38.8
B-05-10	61.9	126.8	37.2	166.9	38.1	60.5			62.8	127.7	38.7	166.2	39.8
B-0.5-20	55.9	132.1	40.6	167.2	42.2	61.9			63.1	132.4	41.2	166.7	42.0
B-0.5-80	90.4					87.1			90.9				
PMMA	111.5					107.3			112.7				

**Table 5:** Isothermal crystallization at 125 °C of extruded PLA and blends: *t_min_*–time of beginning crystallization, *t_max_*–time of maximum heat flow, *t_end_*–time of process completion, *t*_50%_—half-time of crystallization. The heat of crystallization *H_c_* was calculated taking into account the mass of PLA in the sample. Also presented are the values of the long period (*LP*) and the thickness of the crystalline phase (*L_c_*), measured in the SAXS experiment, when determining *L_c_* using the correlation function.

Material	DSC					SAXS	
	t_min_ [min]	t_max_ [min]	t_end_ [min]	t_50%_ [min]	H_c_ [J/g]	LP [nm]	L_c_ [nm]
PLA-i	2	25	69	25	42.6	18.8	4.7
B-i-10	3	52	119	51	44.3	20.8	4.6
B-i-20	7	67	138	66	49.6	22.7	4.8
B-i-80	0	30	104	35	8.9	-	-
PLA-5	1	13	37	12	45.0	18.5	4.9
B-5-10	2	31	69	31	47.3	19.6	4.8
B-5-20	5	42	92	42	47.8	19.6	4.4
PLA-0.5	1	14	33	14	40.9	18.2	6.0
B-0.5-10	2	44	91	45	45.0	21.2	5.1
B-0.5-20	7	54	117	53	50.3	22.7	4.6
B-0.5-80	0	17	95	24	12.0	-	-

## Data Availability

The original contributions presented in this study are included in the article. Further inquiries can be directed to the corresponding author.
